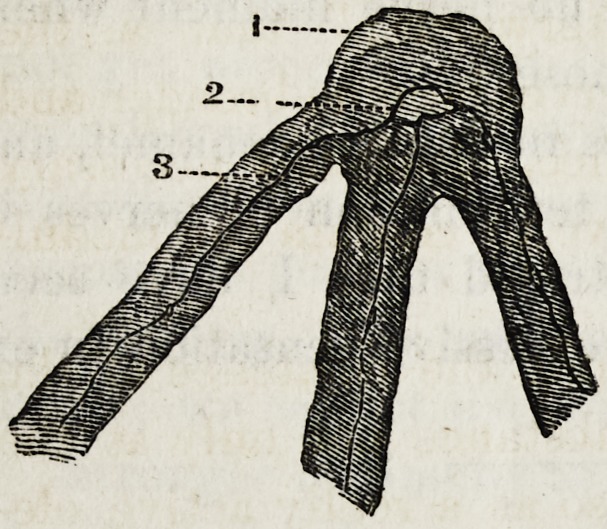# Micrology.—No. 2

**Published:** 1879-05

**Authors:** George B. Harriman

**Affiliations:** Boston, Mass.


### ARTICLE IV.

### *Department of Micrology.*—No. 2.

BY GEORGE B. HARRIMAN, D. D. S., M. D., BOSTON, MASS.

It is now about ten years since I first found nerve filaments within the soft-solid or fibrous portion of the dentine. In 1870 I wrote out what I found to be the facts, and they were published in the *Dental Cosmos*, January, 1871.

Further investigation in 1871 reaffirmed my examinations, and I published them in the *Dental Register*, October, 1872.

In that article, page 400, you will find there cut number 4. I did not state how it had been treated, only that it had been engraved from a photograph of my own taking. It was a thin section of a tooth ground down thin enough to be examined with a 1-50 objective, and treated with 4 per cent. solution of the chloride of gold. See cut number 1 this article.

Figure 1 points to the soft solid substances, in the centre of which can be seen a small nerve filament. Figure 2 points to the lime salts.

In all of my examinations at that time I used the 1-50 of an inch objective, and since then I have used the 1-75 inch objective; a glass that has been pronounced by the most scientific experts in microscopy as exceedingly wonderful in definition, and defining power to verify my observations. In 1872 I wrote as follows: "it is a well-known fact to histological observers that the medulla does not appear until afterwards;" that is, the gray substance of Schwan, when nerves become broader and more easily defined. It is not, hence, absolutely necessary for a constituent of a sensitive nerve to contain the medullary sheath.

The individuals who do not admit the existence of the axis cylinder, regard the white substance not only as the predominating constituent, but also as a really active element of the nerve contents. Prof. Cutter, of New Orleans Dental College, regards the whole of the soft, solid substances as nerve fibres. By making a chemical examination of the entire substance in the so called "tubuli," the result is a large amount of the medullary substance, and that there scarcely exists a tissue rich in cells where this substance does not occur in large quantity.

Still it is only in the nerve fibres that we observe the peculiarity of this substance as such, whilst in all other cellular elements it is contained in finely divided state in the interior of the cells, and is only let free as the contents undergo a change, and are subject to the action of chemical reagents. From blood cells, pus corpuscles, epithelium cells of the most versatile granular parts; from the interior of the spleen and similar organs, unprovided with excretory ducts, this substance can in every case be obtained by extraction. Hence, it is manifest that the medulla can not be the constituent in which reposes the function of the nerve, as such. This same conclusion is arrived at by physical investigations at the present time.

Therefore, the axis cylinder is very generally regarded as the *real, essential constituent of the nerves*, whilst in the white substance it can be only isolated separations of the investing medullary sheath. The *axis cylinder* would hence seem to be the *real electrical substance*, and we may certainly admit the hypothesis, that the medullary sheath rather serves as an isolating mass, which confines the electricity within the nerve itself, and allows its discharge at the non-medullated extremities of the fibres.

Cut No. 2 of this article was taken from a thin shaving turned off from a soft, sensitive molar tooth at the junction of the dentine and enamel, and the lime salts dissolved with mineral acid, and then treated with a solution of the chloride of gold, and is highly magnified. Figure 2 points to the cell ganglion and union of the three nerve axis cylinders, which probably run from the pulp.

Figure 3 points to one of the nerve filaments. It will be observed that the junction of the fibres is made at the enamel.

In a few instances with the use of the 1-50 objective and eye piece micrometer, I have been able to measure some of these nerve fibres, and I am sure that some of them do not measure more than (1-50,000) one-fifty thousandth of an inch in diameter, and it is my opinion that it is only the axis cylinder without the gray substance, that we find in the soft-solids of the dentine.

I now propose to say something in regard to the terminations of nerve fibres. I have given great attention to this subject, especially as they are observed in the fibres of the dentine.

There are several forms of termination given to nerves. Do they end in points terminating in the tissues or cells of the part? or do they end in loops? or are they plexuses? In the teeth we find the nerve fibres through the soft-solid substance towards the enamel and cementum, and in many instances they can be traced entering into its substance, and at their termination they draw together and unite by anastomosis. I do not believe that they *terminate* by *free* extremity. This hypothesis confirms the position, that all nerve fibres form a complete circuit for the current of the nerve force, whether it be sensor or motor. My investigations of the nerves in the teeth go to show that they are continuous, or that you will find no nerve filament where the dentinal fibres do not anastomose.

This termination of nerve fibres in dentine, enamel, and cementum, this ganglion or cell termination of nerves in teeth, was never before demonstrated that I have seen. Have we not some cause for the excessive sensation in excavating teeth.

---

## Figures and Tables

**Figure f1:**
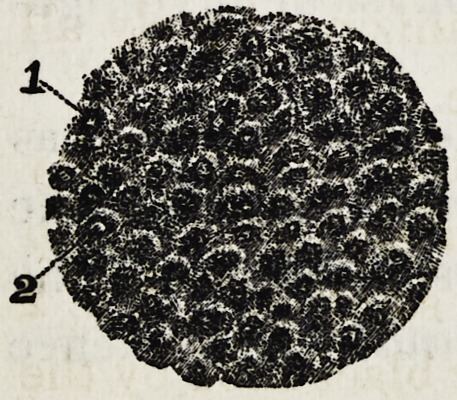


**Figure f2:**